# CircRNA circ_0000190 inhibits the progression of multiple myeloma through modulating miR-767-5p/MAPK4 pathway

**DOI:** 10.1186/s13046-019-1071-9

**Published:** 2019-02-06

**Authors:** Yashu Feng, Ling Zhang, Jieying Wu, Bijay Khadka, Zhigang Fang, Jiaming Gu, Baoqiang Tang, Ruozhi Xiao, Guangjin Pan, Jiajun Liu

**Affiliations:** 1 Department of Hematology, The Third Affiliated Hospital of Sun-yat Sen University, 600 Tianhe Avenue, Guangzhou, 510630 People’s Republic of China; 2 Guangzhou Institutes of Biomedicine and Health, Chinese Academy of Sciences, 190 Kaiyuan Avenue, Guangzhou, 510630 People’s Republic of China

**Keywords:** Circular RNA, Micro RNA, MAPK4, circ_0000190, Multiple myeloma

## Abstract

**Background:**

Multiple myeloma (MM) accounts for 10% of all hematological malignancies. Dysregulation of microRNAs (miRNAs) or long non-coding RNAs (lncRNAs) has important impacts on progression of MM. Circular RNAs (circRNAs) are correlated with malignancy in the modulation of tumor progression. This study aims to investigate the effect of circ_0000190 on regulating the progression of MM.

**Method:**

Microscopic examination via single molecule fluorescent in situ hybridization indicates the location of circ_0000190. qRT-PCR and Western blot were used to evaluate the expression of RNAs and proteins. Potential target of circ_0000190 was searched as miRNA, and examined by luciferase reporter assay. A computational screen was also conducted to search the potential target of miRNA. In vitro cell viability, proliferation, apoptosis assays and flow cytometric were performed to assess the effects of circ_0000190 and its target on MM. Mice model of human MM was established with subcutaneous xenograft tumor, qRT-PCR and western blot were performed to detect the underlying mechanisms of circ_0000190 on MM.

**Results:**

Circ_0000190 was located in the cytoplasm, and down-regulated in both bone marrow tissue and peripheral blood, while the target of circ_0000190, miR-767-5p, was up-regulated, suggesting a negative correlation between them. The binding ability between circ_0000190 and miR-767-5p was confirmed by luciferase reporter assay. Moreover, circ_0000190 inhibited cell viability, proliferation and induced apoptosis of MM thus inhibiting cell progression, which is partially through the negative regulation of miR-767-5p. Mitogen-activated protein kinase 4 (MAPK4) is a direct target of miR-767-5p. In addition, over-expression of miR-767-5p promoted cell progression by directly targeting and regulating MAPK4. The MM model mice with administration of circ_0000190 suppressed tumor growth and progression.

**Conclusion:**

Our results revealed that the ability of circ_0000190 to protect against MM was inherited through repression of miR-767-5p, and miR-767-5p might be a tumor drive through targeting MAPK4. Therefore, a novel role of circ_0000190 on regulating the progression of MM was found, and the clinical application of circRNAs might represent a strategy in MM.

**Electronic supplementary material:**

The online version of this article (10.1186/s13046-019-1071-9) contains supplementary material, which is available to authorized users.

## Background

Multiple myeloma (MM) is a hematological malignancy [1], characterized by multifocal proliferation of plasma cells within the bone marrow (BM) with no initially symptoms [2, 3]. As the second most common hematological cancer, MM accounts for 10% of all hematological malignancies [4]. Although therapeutic strategies have been developed and widely used, the survival rate of MM is still unsatisfactory [3] due to extremely high rate of metastasis, progression and drug resistance [5]. Therefore, the primary task of improving MM prognosis is to study the pathogenesis and search effective therapeutic targets.

Circular RNA (circRNA) is a novel type of non-coding RNA, which widely exists in mammalian cells [6]. The important characteristic of circRNA rests with tissue/cell-type specificity and highly stability to be a biological marker [7–10]. Generally, circRNAs act as competitive endogenous RNAs (ceRNAs) or microRNA (miRNA) sponges, competing for miRNA binding and affecting miRNA function [11, 12]. Some circRNAs can regulate gene expression [13] and modulate transcription [14]. Additionally, emerging evidence have suggested that abnormal expression of circRNAs occurred in various diseases, such as esophageal squamous cell carcinoma, gastric cancer and pancreatic ductal adenocarcinoma [15, 16], suggesting that circRNAs may be closely related to the occurrence and development of tumors. Studies have found that there are thousands of circRNAs transcripts in tumor cells, accounting for a considerable number of total transcripts, indicative the potential ability of circRNAs as novel biomarkers and therapeutic targets for cancer diagnosis and treatment [17–22].

Circ_0000190 is located in human chromosome chr1:224553580–224,559,125 [23]. Previous study has found that circ_0000190 was down-regulated in gastric cancer tissues, and its expression level was closely related to tumor size and metastasis [23]. Since circRNAs are considered as ceRNAs to regulate miRNA action on target gene, and the expression of miR-767-5p was up-regulated in MM [24], we speculated that circ_0000190 may regulate the development of MM through targeting miR-767-5p.

Different signal pathways are involved in the development and drug-resistance of MM, including PI3K/AKT/mTOR, RAS/RAF/MEK/ERK, JAK/STAT, WNT/β-catenin and NF-κB [25].The binding of MM cells to BM stromal cells triggers adhesion- and cytokine-mediated MM cell growth, survival and migration through activation of p42/p44 MAPK [26]. Silencing of IL-16 using siRNA reduced the proliferation of end-stage myeloma cells through MAPK and PIK3 pathways [27, 28]. p38 MAPK shRNA-treated MM cells significantly restored the generation of osteoclasts by the addition of DKK-1 and MCP-1 [29]. Down-regulation of ERK/MAPK and NF-κB significantly slowed down the myeloma growth in subcutaneous xenograft mouse models [30]. All these researches confirmed the vital relation between MAPK and MM. However, the mechanism of circ_0000190/miR-767-5p/MAPK4 in MM is not clear up to now.

In this study, we firstly investigated the effect of circ_0000190 on MM,and characterized the functional downstreammiR-767-5p and MAPK4. We also investigated the roles of circ_0000190, miR-767-5p and MAPK4 in the progression of MM, and further explored their potential mechanisms. The present study was also undertaken to evaluate the biological roles of circ_0000190 in experimental mice model of human MM established with subcutaneous xenograft tumor. Evidence showed that circ_0000190 suppressed in vivo tumor growth and progression. The findings reported in this study are of tremendous clinical significance due to the clarification of the impact of circ_0000190 on miR-767-5p/MAPK4 could serve as a basis for the development of predictive biomarkers of tumor emergence and prevention in MM.

## Materials and methods

### Patients and samples collection

Approval for this study was obtained from the Ethics Committee of the The Third Affiliated Hospital Sun-yat Sen University. Written informed consents were obtained from all participants involved in the study before using these clinical samples for research purposes. All bone marrow tissues and peripheral blood specimens were obtained from patients pathologically and clinically diagnosed as MM or normal people (volunteers). The diagnosis, stage and risk status of MM were made in accordance with the National Comprehensive Cancer Network. The general clinical and laboratory features of the patients are summarized in Table 1.

**Table 1:** Difference in the circRNA_0000190 expression in multiple myeloma patients grouped by clinicopathological characteristics

Clinicopathological characteristics	Number of patients	Expression of circRNA_0000190 in tissues^a^	*P*- value
Gender			
Male	21	0.74 ± 0.08	0.192
Female	26	0.58 ± 0.08	
Age (year)			
< 42	24	0.64 ± 0.08	0.692
≥ 42	23	0.68 ± 0.09	
M protein			
IgG	14	0.66 ± 0.08	0.878
IgA	13	0.61 ± 0.11	
Light chain	20	0.68 ± 0.10	
ISS stage			
I	8	1.10 ± 0.17	**< 0.0001**
II	16	0.78 ± 0.08	
III	23	0.42 ± 0.04	
Durie-Salmon stage			
I	7	1.37 ± 0.09	**< 0.0001**
II	10	0.85 ± 0.07	
III	30	0.43 ± 0.03	
Hypercalcemia			
With	15	0.75 ± 0.11	0.297
Without	32	0.62 ± 0.07	
Renal insufficiency			
With	16	0.71 ± 0.12	0.496
Without	31	0.63 ± 0.06	
Anemia			
With	38	0.63 ± 0.06	0.261
Without	9	0.79 ± 0.13	
Bone disease			
With	29	0.66 ± 0.08	0.81
Without	18	0.66 ± 0.08	
Cytogenetic abnormality			
With	12	0.73 ± 0.14	0.468
Without	36	0.63 ± 0.06	

^a^ The relative expression of circRNA_0000190 was calculated using 2^−∆∆Cq^ method and was shown as mean ± SE

### Single-molecule RNA fluorescence in situ hybridization (smFISH)

To detect the intracellular location of circ_0000190, the RNA-FISH procedure was performed as described in Biosearch Technologies (https://www.biosearchtech.com; Petaluma, CA) and the fluorescence-conjugated circ_0000190 probes were obtained from Guangzhou Vipotion Biotechnology Co.,Ltd. (Guangzhou, China). Briefly, cells were rinsed in PBS and fixed in 4% formaldehyde solution with RNase-free PBS for 10 min at room temperature. Cells were then incubated with 0.1% Triton X-100 solute in PBS on ice for 5 min. Fluorescence-conjugated circ_0000190 probes were hybridized with the samples for 4 h in the dark at 37 °C. Laser scanning confocal microscopy (Carl Zeiss, Jena, Germany) was used to visualize the samples described above.

### Cell culture

The MM cell lines MM.1S, NCI-H929, were purchased from American Type Culture Collection (ATCC, Manassas, VA, USA).The cell lines are cultured in RPMI1640 medium containing 10% FBS (GIBCO, Life Technologies, Carlsbad, CA, USA), with additional 2 μM glutamine, 100 U/ml penicillin and 100 μg/mL streptomycin (GIBCO). The 37 °C constant temperature incubator was used to incubating cells with 5% CO_2_.

### RNA preparation and qRT-PCR

Sample RNAs were extracted by the means of Trizol reagent (Invitrogen, Carlsbad, CA, USA). The cDNAs were synthesized using the Reverse Transcription System Bestar qPCR RT Kit according to the manufacture instruction with ABI 7500 Real-Time PCR System (Applied Biosystems, Lincoln Centre Drive Foster City, CA 94404, USA). U6 was used as the internal reference and GAPDH as the endogenous controls. The fold change was determined using 2^−ΔΔCt^ (where ΔCt = (Ct of target of interest) − (Ct of endogenous control gene), ΔΔCt = (ΔCt of target of interest) − (ΔCt of endogenous control gene). Three technological replicates were used to ensure the reliability of the analysis. The primer sequences were showed: circ_0000190, 5’-GGTTTCCACTTGCTCTGCTT-3′ (forward) and 5’-CAGTGCAATGACATGAGCAGTA-3′ (reverse); miR-767-5p, 5’-CTCAACTGGTGTCGTGGAGTCGGCAATTCAGTTGAGCATGCTCAG-3′ (forward) and 5′- ACACTCCAGCTGGGTGCACCATGGTTGTCTGAG-3′ (reverse); U6, 5’-CTCGCTTCGGCAGCACA-3′ (forward) and 5’-AACGCTTCACGAATTTGCGT-3′ (reverse); MAPK4, 5’-TGAGAAGGGTGACTGCATCG-3′ (forward) and 5’-ACCAAACCATTGACACCGAAG-3′(reverse); GAPDH, 5’-TGTTCGTCATGGGTGTGAAC-3′ (forward) and 5’-ATGGCATGGACTGTGGTCAT -3′(reverse).

### Plasmid construction and transfection

For the ectopic expression of circ_0000190, the synthetic circ_0000190 sequence was subcloned into the pcDNA3.0 vector (Invitrogen). PCR was used to get the sequence of MAPK4 with the following primer sequences: 5’-CCCAAGCTTATGGCTGAGAAGGGTGACTGCATC-3′ (forward) and 5’-CCGCTCGAGTCACAGGGTACCAGCAAAGAGCATT-3′ (reverse). Expression plasmids pcDNA3.0 (Invitrogen) were then constructed and sequenced for the ectopic expression of MAPK4. Short hairpin RNAs (shRNA) for circ_0000190 (5’-ACCAAAGCATCTAGTGCTTTT-3′) and MAPK4 (5’-GAAGCTCTCCAGACCATTT-3′) were synthesized by GenePharma Co., Ltd. (Shanghai, China) and utilized for cell transfection. A negative control (NC, 5’-UUCUCCGAACGUGUCACGUTT-3′) was also used. For the gain- and loss-of-function experiments, 100 μL 10,000 MM.1S, NCI-H929 cells of each group per well were cultured in 6-well plates for 24 h, and then 100 pM miRNA mimics or inhibitor of miR-767-5p (Invitrogen), 40 nM the abovementioned plasmids or shRNAs were transfected into cultured cells with lipofectamine 2000 (Invitrogen) according to the manufacturer’s instructions.

### Target prediction and luciferase assay

By the means of starbase (http://starbase.sysu.edu.cn), we successfully predict binding sites between circ_0000190 and miR-767-5p, with TargetScan (http://www.targetscan.org) website to explore the putative targets of miR-767-5p. We also construct circ_0000190 wild-type and mutant 3’UTR reporter vectors with the primer: 5’-CCGCTCGAGTAATTCCAGAATTGATTGGCCATA-3′ (forward) and 5’-ATTTGCGGCCGCCTATAAAGATACATGAAGAAGCAGAGC-3′ (reverse), (5’-ATATATCGATACATTCGCTCAGCGAGTGGTAACATGG-3′ (forward) and 5’-CCATGTTACCACTCGCTGAGCGAATGTATCGATATAT-3′ (reverse) respectively and subcloned into the psiCHECK2pRL-TK (promega, Madison, USA). MAPK4 wild-type (5’-CCGCTCGAGGACAACAAGCCGCACCACTACTC-3′ (forward) and 5’-GGGTTTAAACGGTGCTGTGATGTGAGGGTGAAC-3′ (reverse)), mutant-1 (5’-AAACTGGGCGACCTCATAACGCTGTGCATCCCCGAGCA-3′ (forward) and 5’-TGCTCGGGGATGCACAGCGTTATGAGGTCGCCCAGTTT-3′ (reverse)), mutant-2 (5’-AGGGGAGACCACATGGCAGTAACAGGGAAGAAACGG-3′ (forward) and 5’-CCGTTTCTTCCCTGTTACTGCCATGTGGTCTCCCCT-3′ (reverse)) and mutant-3 (5’-AGGCTAAGGTGAGTTGCCATGATGCAAACCTGTGTG-3′ (forward) and 5’-CACACAGGTTTGCATCATGGCAACTCACCTTAGCCT-3′ (reverse)) primers were also used. Each plasmids together with miR-767-5p mimics or a negative control mimics were transfected into MM cells. Firefly luciferase gene in the vector psiCHECK2 was used as a control for transfection efficiency. By dual-luciferase assay reporter kit and lumat LB 9501 luminator, we detect the Firefly luciferase and Renilla luciferase signals.

### Cell proliferation assay

100 μL 8000 MM.1S, NCI-H929 cells of each group per well were cultured in 96-well plates for 24 h and then were cultured for 24, 48 and 72 h. At each time point, the CCK-8 assay (Solarbio, Beijing, China) was used to evaluate cell viability upon cells according to the manufacturer’s protocol. Cell viabilities were calculated by measuring the optical density at 450 nm, using a spectrophotometric plate reader (BioTek, VT, USA). All cell viability results were tested by three independent experiments.

200 μL 2 × 10^4^/mL cultured MM cells were fixed with 70% alcohol and then incubated with 50 μM EdU (5-Ethynyl-2′-Deoxyuridine) labeling solution (Invitrogen) at 37 °C for 2 h. According to the Click-iT™ EdU imaging kit (Invitrogen), the fluorescent intensity of EdU was determined at 550 nm. For subsequent DNA staining, cells were incubated with 5 μg/mL Hoechst 33342 for 30 min. Immunostainings were visualized and photographed with a fluorescent microscope (Olympus inverted microscope IX71) and calculated by flow cytometry.

### Flow cytometry

3.5 × 10^3^ cells/well MM.1S and NCI-H929 cells treated with different experimental conditions were harvested, washed with PBS and then fixed with 70% ethanol at 4 °C for 30 min. Upon washing with PBS, the supernatant was removed. The resulting cells were then treated with ribonuclease (Abcam, Cambridge, MA, USA), followed by the addition of RNase (50 μL, 100 μg/mL, Abcam) and then the propidium iodide (PI, 200 μL, Abcam) to stain the DNA. Cells were analyzed with a FACS flow cytometer equipped with an excitation laser at 488 nm. The PI was collected through a 605 nm band pass filter.

For cell apoptosis detection, In Situ Cell Death Detection Kit, Fluorescein (Sigma) was utilized. According to the manufacturer’s instructions, 4 × 10^3^ MM cells were fixed with 4% formaldehyde fixative buffer and incubated for 20 to 30 min at room temperature. After removing the fixative, cells were washed with PBS for 2–3 times. Then 50 μL reaction mixture were added and incubated at 37 °C for 60 min. Remove the reaction mixture, and wash the cells 3–5 times with 200 μL/well of PBS. The fluorescence intensity was monitored by flow cytometer at excitation wavelength of 550 nm and emission wavelength of 565 nm.

### Western blot

We collected and flash-freezed the cells by liquid nitrogen, ultrasonic cell-break method was adopted to break the cell wall, with 5 s in 50 mM lysis buffer (20 mM Tris pH 7.5, 150 mM NaCl, 1 mM PMSF, 10 mM β-glycerophosphate, 1% Triton X-100, 5 mM EDTA, 0.2 mM Na_3_VO_4_, 2 μg/mL leupeptin, 2 μg/mL pepstatin A) on ice for twice. Homogenates were centrifuged at 12000 g at 4 °C for 30 min, then the supernatants were collected. Protein lysates (30 μg) were loaded onto the sodium dodecyl sulfate-polyacrylamide gels (SDS-PAGE) for electrophoresis and transferred to polyvinylidene fluoride (PVDF) membranes. After blocking in PBS-T with 5% BSA for 1 h, PVDF membranes were incubated overnight with the primary antibody as follows: monoclonal antibodies against MAPK4 (1:1000, Santa Cruz, CA, USA), CDK4 (1:2000), CDK6 (1:5000), cyclin D1 (1:5000), cyclin E (1:2000), p21 (1:2000). Washed with TBST (10 min × 3 times), the membranes were then probed with the appropriate secondary antibody (1:5000; Abcam). Immunoreactivity was determined and observed using enhanced chemiluminescence (Millipore, Billerica, MA,USA). GAPDH was used as a control.

### Immunofluorescence

MM.1S and NCI-H929 cells were fixed in PBS with 4% paraformaldheyde and permeabilized in 0.1% Triton X-100. After blocking with PBS containing 5% BSA, cells were incubated overnight at 4 °C with the following primary antibodies: anti-MAPK4 (Abcam) followed by the incubation with the proper secondary antibody fluorescein isothiocyanate (FITC)-conjugated for 30 min. Cells were then washed with PBS and nuclei stained by DAPI (Sigma). Cells were observed and photographed under aFV10i Confocal microscope (Olympus, Japan).

### Animal experimental model

All studies involving animals were conducted in accordance with the guidelines set out by the Sun-yat Sen University Institute Animal Care and Use Committee. The mice were housed in a pathogen-free animal facility at Sun-yat Sen University School of medicine animal facilities separately with standard pellet diet and water. Female NOD.CB17-*Prkdc*^scid^/NCrHsd (nonobese diabetic /serious combined immunodeficiency disease, NOD/SCID; Harlan Laboratories, Indianapolis, IN) mice 4–6 weeks of age were implanted subcutaneously with 1.2 × 10^7^ MM.1S or NCI-H929 cells. Treatment was also initiated at the first subcutaneous injection with MM.1S or NCI-H929 cells transfected with either pcDNA3.0-circ_0000190 or shRNA-circ_0000190, ten mice in each group. Tumors were measured twice weekly with digital calipers and tumor volume was calculated using the equation: tumor volume (mm^3^) = L × W^2^ × 0.5; where length (L) is the longest diameter of the tumor and W (width) is the smaller diameter. Mice were euthanized by ether inhalation at the termination of the study. Tumors were excised and fixed in formalin for other experiments.

### Statistical analysis

All data are presented as mean ± SEM. All experiments were performed at least in three independent times. By the means of one-way analysis of variance (ANOVA) followed by Duncans multiple-comparison test using SPSS 19.0 (SPSS Inc., Chicago, IL, USA), we calculated the statistical significance. *P* <  0.05, *P* <  0.01 or *P* <  0.001 was regarded as statistically significant.

## Results

### Circ_0000190 is down-regulated in MM tissues and peripheral blood

**Fig. 1:**
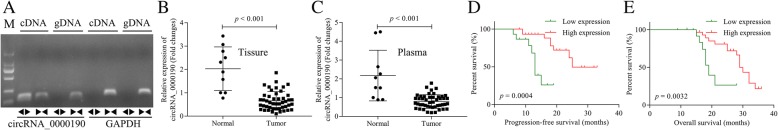
Circ_0000190 is down-regulated in MM tissues and peripheral blood. **a** Divergent primers detected circular RNAs in cDNA but not gDNA. **b** RT-PCR analysis of circ_0000190 in human MM and normal healthy tissues. Tumor vs. Normal, *P* < 0.001. **c** qRT-PCR analysis of circ_0000190 in human MM and normal healthy peripheral blood. Tumor vs. Normal, *P* < 0.001. **d** Progression-freecurve analysis of high or low expression of circ_0000190 in MM patients. **e** Overall survival curve analysis of high or low expression of circ_0000190 in MM patients

Circ_0000190 was first confirmed via PCR in both cDNA and genome DNA (gDNA) (Fig. 1a), as PCR with gDNA yielded no products while cDNA yielded completely consistent sequences for circ_0000190. According to RNA-FISH results, we firstly found that circ_0000190 was mainly located in the cytoplasm of MM cells (Additional file 1: Figure S1), indicating that circ_0000190 may exert its biological function in the cytoplasm. We then determined the circ_0000190 expression level in human MM tissue and peripheral blood. A significantly down-regulation of circ_0000190 was found in both tissue (*P* <  0.001) (Fig. 1b) and plasma (*P* < 0.001) (Fig. 1c) samples in comparison to normal groups. These data confirmed the vitally important relation between circ_0000190 and MM. We then made a thorough inquiry of survival curve analysis to show the summary display of survival rates over time in MM patients. Interestingly, the survival data showed a lower risk for patients with higher expression of circ_0000190, where the higher expression of circ_0000190 with the longer progression-free (PFS) (Fig. 1d) and overall survival (OS) (Fig. 1e) survival time. These data suggested thatcirc_0000190 predicts excellent clinical outcome in patients with MM. We then did the statistics of the clinical characteristics of MM patients. As shown in Table 1, International staging system (ISS) stage I and durie-salmon (DS) stage I shared significantly higher expression of circ_0000190 compared to stage II and III. While the others such as gender, age, M proteins, renal insufficiency shared no significant difference in the expression of circ_0000190. This suggests that the stage of ISS and DS was correlated with the expression of circ_0000190, and circ_0000190 was down-regulated in patients with MM. Taken together, circ_0000190 is down-regulated in MM tissues and plasma.

### Circ_0000190 inhibits the progression of MM

**Fig. 2:**
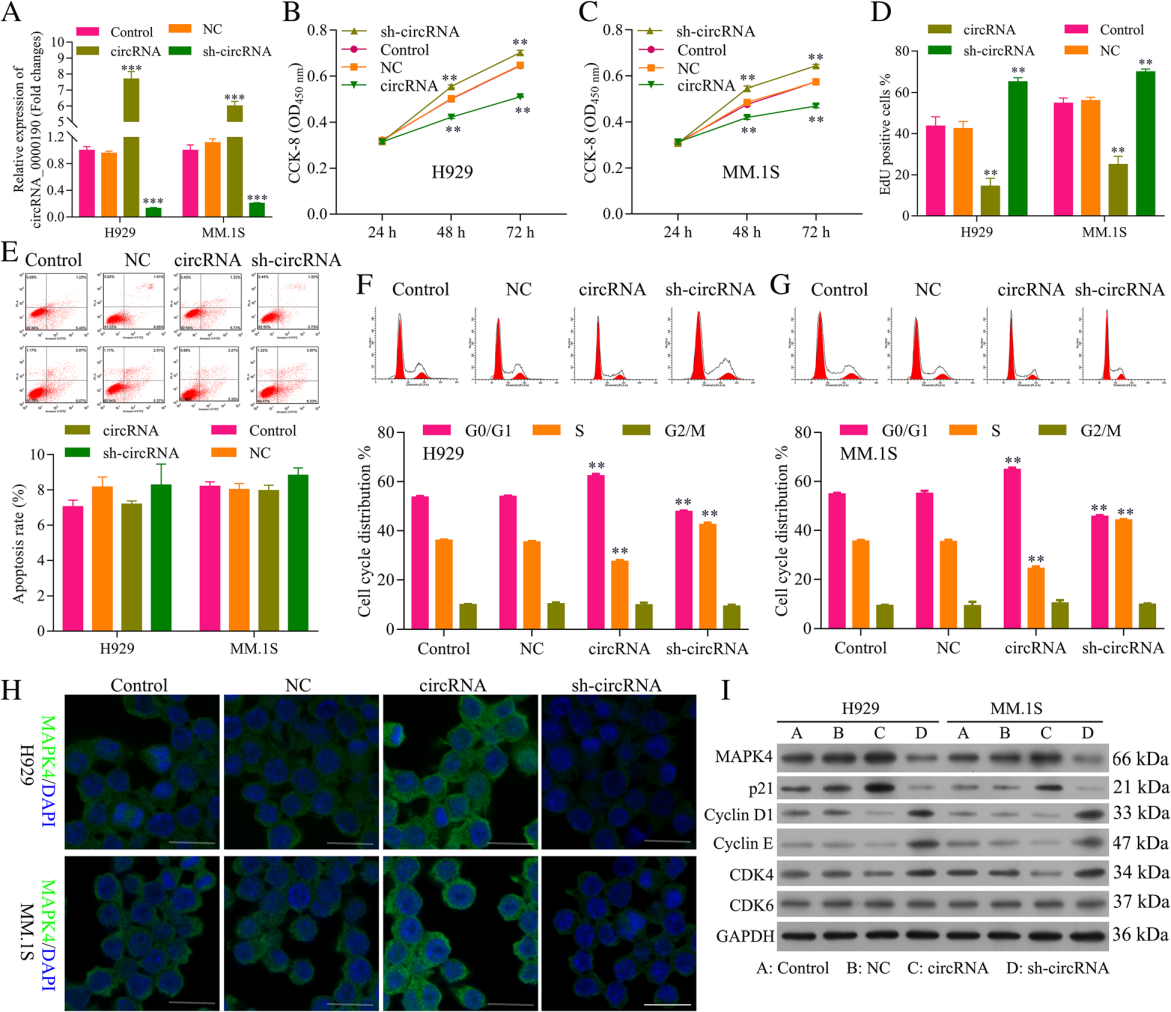
Circ_0000190 inhibits the progression of MM. **a** Transfection efficiency of circ_0000190over-expression and knocking down were determined by qRT-PCR. *** circRNA or sh-circRNA vs. control, *P* < 0.001. **b** The CCK8 assay detects the effect of circ_0000190and sh-circ_0000190on cell viability of H929 cells. ** circRNA or sh-circRNA vs. control, *P* < 0.01. **c** The CCK-8 assay detects the effect of circ_0000190 and sh-circ_0000190 on cell viability of MM. 1S cells. ** circRNA or sh-circRNA vs. control, *P* < 0.01. **d** EdU labeling assay detects the effect of circ_0000190and sh-circ_0000190 on cell proliferation. ** circRNA or sh-circRNA vs. control, *P* < 0.01. **e** Flow cytometry apoptosis method detects the effect of circ_0000190 and sh-circ_0000190 on cell apoptosis. **f** Flow cytometry detects the effect of circ_0000190 and sh-circ_0000190 on cell cycle of H929 cells. ** circRNA or sh-circRNA vs. control, *P* < 0.01. **g** Flow cytometry detects the effect of circ_0000190 and sh-circ_0000190 on cell cycle of MM. 1S cells.** circRNA or sh-circRNA vs. control, *P* < 0.01. (H) IF staining detects the effect of circ_0000190 and sh-circ_0000190 on the expression of MAPK4. **i** Western blot analysis detects the effect of circ_0000190 and sh-circ_0000190 on the expression of MAPK4, CDK6, CDK4, Cyclin D1 and Cyclin E, p21^Cip1^. **P < 0.01, ***P < 0.001

The basic expression level of circ_0000190 in the MM cell lines MM.1S and NCI-H929 cells have been detected in Additional file 2: Figure S2. The efficiency of plasmids highly expressed circ_0000190 (circ_0000190) and knockdown of circ_0000190 by shRNA (sh-circ_0000190) was tested by qRT-PCR, which showed a significant increment for circ_0000190 (*P* < 0.001) and reduction for sh-circ_0000190 (*P* < 0.001), respectively (Fig. 2a). The CCK-8 assay demonstrated that NCI-H929 (Fig. 2b) and MM.1S (Fig. 2c) cells transfected with circ_0000190 significantly decreased cell viability in comparison to control (*P* < 0.01), while knockdown of circ_0000190 increased cell viability (*P* < 0.01). The effect of circ_0000190 on the proliferation of MM cells was examined by flow cytometry analysis using EdU-labeled probes. Consistent with CCK8 assay, sh-circ_0000190 significantly promoted the proliferation of MM cells while circ_0000190 inhibited the proliferation (*P* < 0.01) (Fig. 2d), as the number of EdU positive cells in cells treated with sh-circ_0000190 was more than in cells with control and even less in circ_0000190. Flow cytometry apoptosis results demonstrated that circ_0000190 did not affect cell apoptosis (Fig. 2e), and Tunel staining demonstrated that sh-circ_0000190 little inhibited cell apoptosis while circ_0000190 little promoted apoptosis (Additional file 3: Figure S3). Flow cytometry of the cell cycle also confirmed that circ_0000190 over-expression inhibited cell cycle and blocks cell cycle at G1 stage, knockdown of circ_0000190 promoted cell cycle progression in both H929 (Fig. 2f) and MM. 1S cells (Fig. 2g). These data suggested that circ_0000190 plays an anti-cancer role in MM with the inhibiting ability of cell proliferation.

We also detect the expression of MAPK4 and proteins involved in the cell cycle. Immunofluorescence (IF) showed that the expression of MAPK4 in MM.1S and NCI-H929 cells treated with circ_0000190 was dramatically increased, while decreased in sh-circ_0000190 (Fig. 2h), in line with the result in Western blot (Fig. 2i). On the other hand, cyclin-dependent kinases (CDKs), a family of serine/threonine kinases, bind to the regulatory protein cyclin and play an important role in regulating the cell cycle and then the progression of cells [31, 32], CDK6-Cyclin D [33], CDK4-Cyclin D [34] complexes are important for the G1 phase progression and G1/S transition of the cell cycle.p21^Cip1^ [35] is cyclin-dependent kinase inhibitor able to inhibiting cyclin/CDK complexes and then cell cycle. Consistent with the results of flow cytometry, circ_0000190 over-expression decreased the expression of CDK4, Cyclin D1 and Cyclin E, while increased p21^Cip1^, thus blocking cell cycle at G1 stage (Fig. 2i). On the other hand, circ_0000190 knockdown increased the expression of CDK4, Cyclin D1 and Cyclin E, while decreased p21^Cip1^, thus promoting cell progression (Fig. 2i), confirming the inhibition ability of cell progression for circ_0000190.

### miR-767-5p is a target of circ_0000190 and promotes the progression of MM

**Fig. 3:**
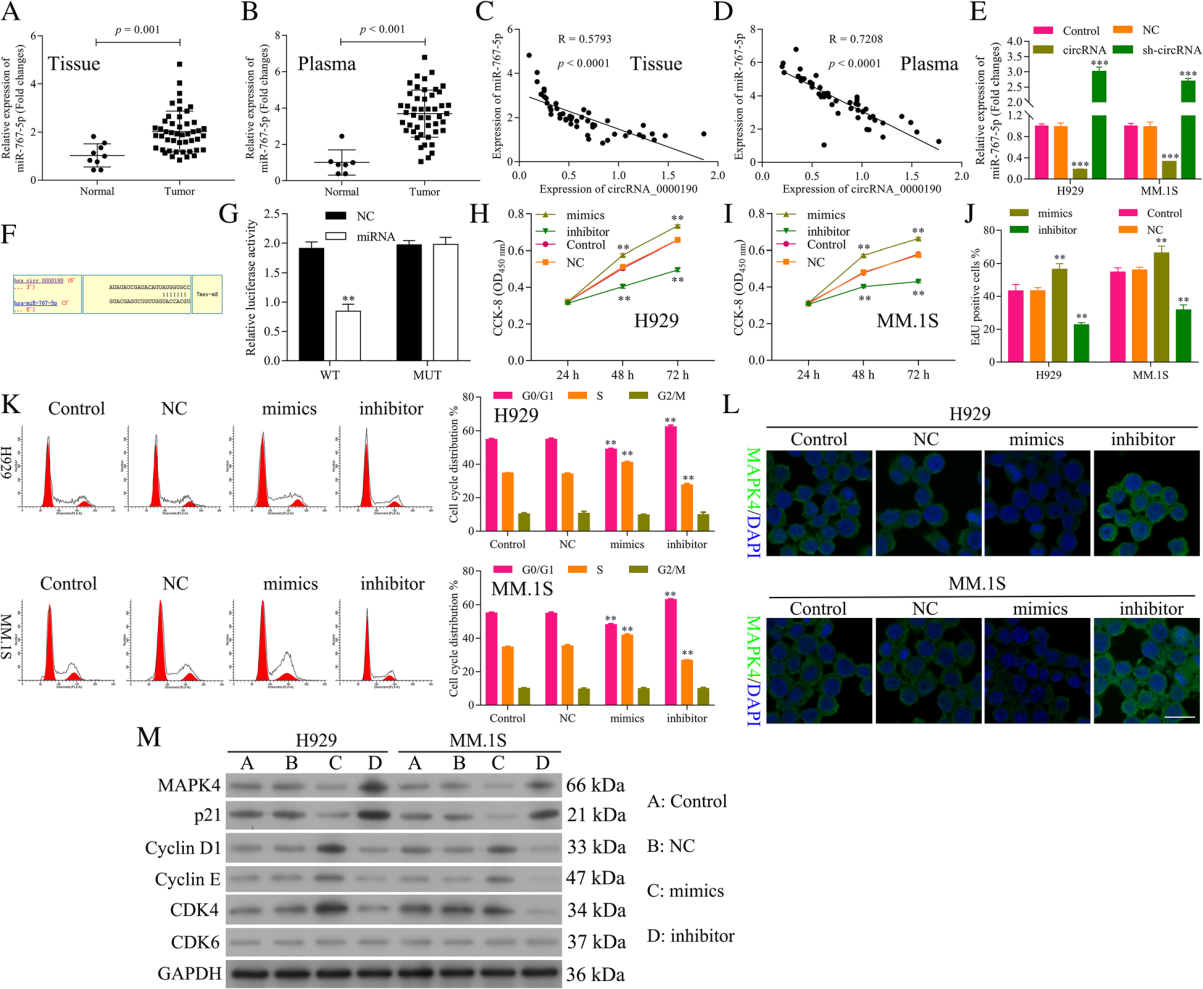
miR-767-5p is a target of circ_0000190 and promotes the progression of MM. **a** qRT-PCR analysis of miR-767-5p in human MM and normal healthy tissues. Tumor vs. Normal, *P* < 0.001. **b** qRT-PCR analysis ofmiR-767-5p in human MM and normal healthy peripheral blood. Tumor vs. Normal, *P* < 0.001. **c** Relativity analysis showed a negative correlation between circ_0000190 and miR-767-5p in human MM tissues. **d** Relativity analysis showed a negative correlation between circ_0000190 and miR-767-5p in human MM plasma. **e** qRT-PCR detects the effect of circ_0000190 and sh-circ_0000190 on the expression of miR-767-5p in MM. 1S and H929 cells. *** circRNA or sh-circRNA vs. control, *P* < 0.001. **f** The predicted binding sites for miR-767-5p in the 3’UTR of circ_0000190 and the mutations in the binding sites are shown. **g** Luciferase reporter assay of circ_00001903’UTR-wild type and mutant with miR-767-5p. ** miR-767-5p mimics vs. NC, *P* < 0.01. **h** The CCK-8 assay detects the effect of miR-767-5p mimics and inhibitor on cell viability of H929 cells. ** miR-767-5p mimics or inhibitor vs. control, *P* < 0.01. **i** The CCK-8 assay detects the effect of miR-767-5p mimics and inhibitor on cell viability of MM. 1S cells. ** miR-767-5p mimics or inhibitor vs. control, *P* < 0.01. **j** EdU labeling assay detects the effect of miR-767-5p mimics and inhibitor on cell proliferation. ** miR-767-5p mimics or inhibitor vs. control, *P* < 0.01. **k** Flow cytometry detects the effect of miR-767-5p mimics and inhibitor on cell cycle. ** miR-767-5p mimics or inhibitor vs. control, *P* < 0.01. **l** IF staining detects the effect of miR-767-5p mimics and inhibitor on the expression of MAPK4. **m** Western blot analysis detects the effect of miR-767-5p mimics and inhibitor on the expression of MAPK4, CDK6, CDK4, Cyclin D1 and Cyclin E, p21^Cip1^. **P < 0.01, ***P < 0.001

MiR-767-5p expression level was significantly evaluated in human MM tissue (Fig. 3a) and plasma (Fig. 3b) compared with normal group (*P* < 0.001). Relativity analysis showed a negative correlation between circ_0000190 and miR-767-5p in both tissue (Fig. 3c) and plasma samples (Fig. 3d), suggesting potential binding ability between them. The expression of miR-767-5p in MM.1S and NCI-H929 cells treated with circ_0000190 was dramatically decreased, while increased in sh-circ_0000190 (Fig. 3e), consistent with the negative correlation analysis. Next we utilized computational prediction in starbase to identify the potential binding sites for miR-767-5p on circ_0000190 (Fig. 3f). The binding ability between miR-767-5p and circ_0000190 was confirmed by luciferase assay. Over-expression of miR-767-5p significantly decreased luciferase activity of reporter gene with wild-type circ_0000190 3’UTR compared with negative control (*P* < 0.01) (Fig. 3g). However, this regulatory effect of miR-767-5p was suppressed when the predicted binding site in 3’UTR of circ_0000190 was mutated (Fig. 3g).

In order to explore the functional role of miR-767-5p on MM, mimics and inhibitor were transfected into MM cell lines. The CCK-8 assay demonstrated that NCI-H929 (Fig. 3h) and MM.1S cells (Fig. 3i) transfected with miR-767-5p mimics significantly increased cell viability, while inhibitor decreased cell viability (*P* < 0.01). EdU staining also showed promotion in the proliferation of MM cells by mimics while inhibition in the proliferation by inhibitor (Fig. 3j). Flow cytometry and Tunel staining results demonstrated that miR-767-5p did not affect cell apoptosis (Data not shown). Flow cytometry of the cell cycle also confirmed that miR-767-5p inhibitor inhibited cell cycle and blocks cell cycle at G1 stage, while mimics promoted cell cycle progression (Fig. 3k). These data suggested that miR-767-5p promotes the cell progression of MM, in contrast to circ_0000190. IF (Fig. 3l) and Western blot (Fig. 3m) showed the down-regulation of MAPK4 by miR-767-5p mimics, while up-regulation by inhibitor. Similar to sh-circ_0000190, miR-767-5p mimics increased the expression of CDK4, Cyclin D1 and Cyclin E, while decreased p21^Cip1^, thus blocking cell cycle at G1 stage (Fig. 3m). miR-767-5p inhibitor decreased the expression of CDK4, Cyclin D1 and Cyclin E, while increased p21^Cip1^, thus promoting cell progression (Fig. 3 m), confirming the promotion ability of cell progression for miR-767-5p.

### Circ_0000190 inhibits progression of MM by inhibiting miR-767-5p

**Fig. 4:**
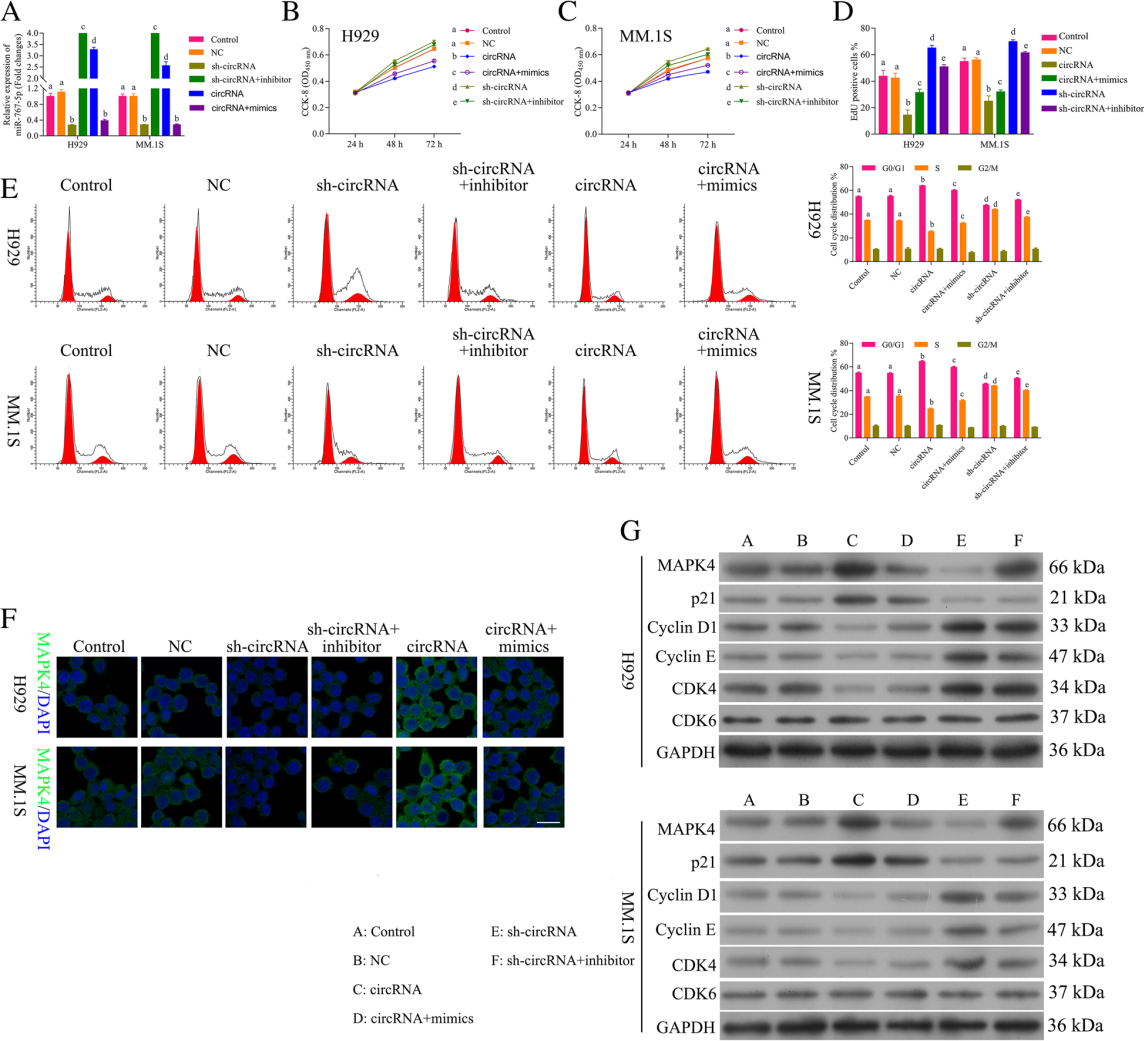
Circ_0000190 inhibits progression of MM by inhibiting miR-767-5p. **a** qRT-PCR detects the effect of circ_0000190 and sh-circ_0000190, miR-767-5p mimics and inhibitor on the expression of miR-767-5p in MM. 1S and H929 cells. **b** The CCK8 assay detects the effect of circ_0000190 and sh-circ_0000190, miR-767-5p mimics and inhibitor on cell viability of H929 cells. **c** The CCK8 assay detects the effect of circ_0000190 and sh-circ_0000190, miR-767-5p mimics and inhibitor on cell viability of MM. 1S cells. **d** EdU labeling assay detects the effect of circ_0000190 and sh-circ_0000190, miR-767-5p mimics and inhibitor on cell proliferation. **e** Flow cytometry detects the effect of circ_0000190 and sh-circ_0000190, miR-767-5p mimics and inhibitor on cell cycle. **f** IF staining detects the effect of circ_0000190and sh-circ_0000190, miR-767-5p mimics and inhibitor on the expression of MAPK4. **g** Western blot analysis detects the effect of circ_0000190 and sh-circ_0000190, miR-767-5p mimics and inhibitor on the expression of MAPK4, CDK6, CDK4, Cyclin D1 and Cyclin E, p21^Cip1^. Different letters in the figure indicate statistically significant differences (*P* < 0.05)

In order to evaluate whether circ_0000190 inhibited progression of MM by specifically targeting miR-767-5p, we examined four groups of MM cell models: circ_0000190 over-expression, circ_0000190 over-expression+miR-767-5p mimics, sh-circ_0000190, sh-circ_0000190 + miR-767-5pinhibitor. qRT-PCR (Fig. 4a) demonstrated down-regulation of miR-767-5p by circ_0000190 while up-regulation by miR-767-5p mimics, up-regulation by sh-circ_0000190 while down-regulation by inhibitor. The cell viability in H929 (Fig. 4b) and MM. 1S (Fig. 4c), as well as cell proliferation (Fig. 4d) assays showed increase by sh-circ_0000190 and decrease by addition of miR-767-5pinhibitor, decrease by circ_0000190 and increase by addition of mimics. Flow cytometry of the cell cycle also confirmed that circ_0000190 inhibited cell cycle and blocks cell cycle at G1 stage, while addition of miR-767-5p mimics promoted cell cycle progression (Fig. 4e). Sh-circ_0000190 promoted cell cycle while inhibited by addition of inhibitor(Fig. 4e). These data suggested that circ_0000190 inhibited progression of MM by inhibiting miR-767-5p. IF (Fig. 4f) and Western blot (Fig. 4g) also confirmed the above conclusion, demonstrated that circ_0000190 increased the expression of MAPK4, while decreased by addition of mimics, sh-circ_0000190 decreased MAPK4 while increased by inhibitor The expression of CDK4, Cyclin D1 and Cyclin E were inhibited and p21^Cip1^ was promoted by circ_0000190, while addition of mimics increased CDK4, Cyclin D1 and Cyclin E and decreased p21^Cip1^, in contrast to sh-circ_0000190 and inhibitor (Fig. 4g).

### MAPK4 is target of miR-767-5p and inhibits progression of MM

**Fig. 5:**
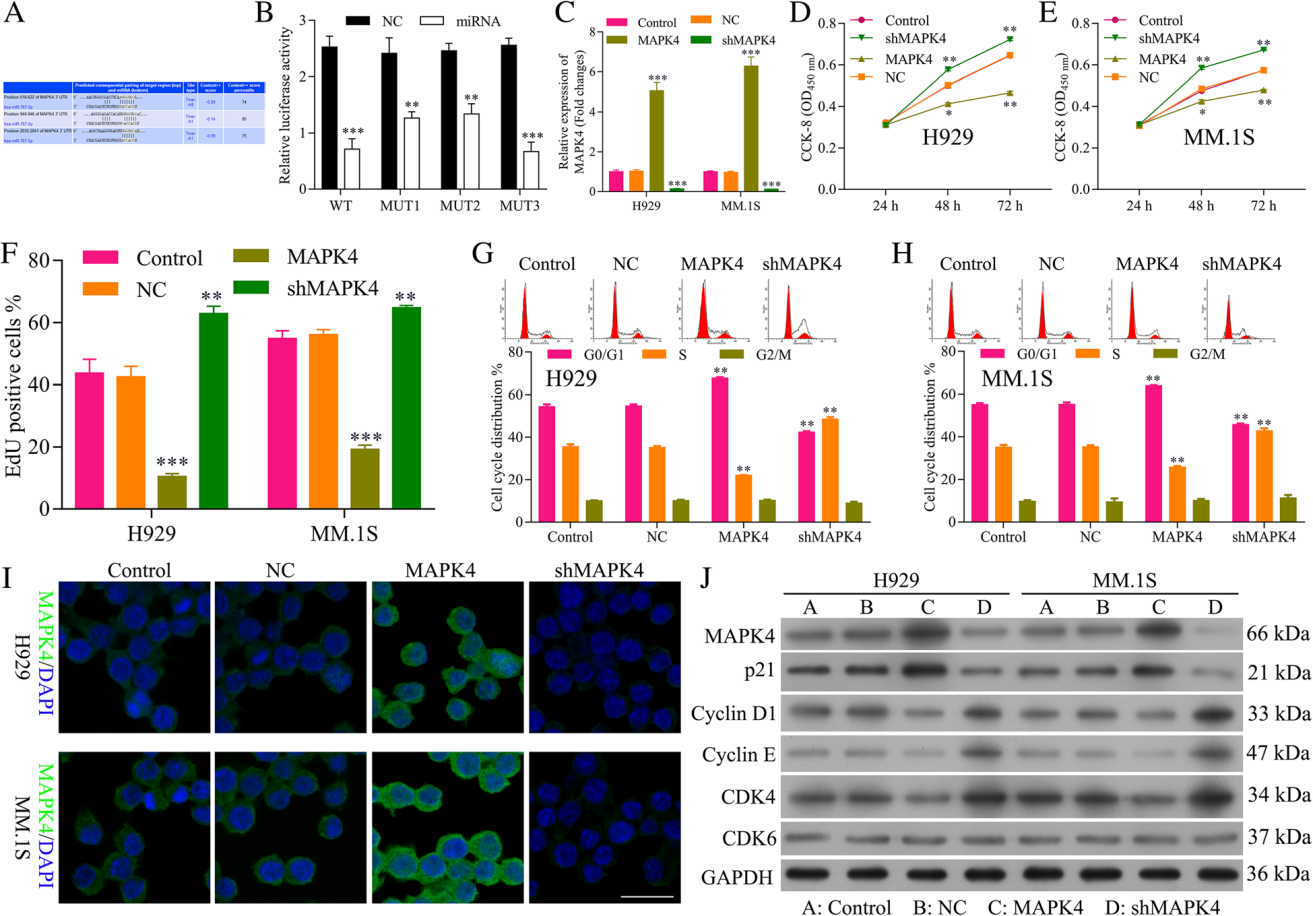
MAPK4 is a target of miR-767-5p and inhibits progression of MM. **a** The three predicted binding sites for miR-767-5p in the 3’UTR of MAPK4 and the mutations in the binding sites are shown. **b** Luciferase reporter assay of MAPK43’UTR-wildtype and mutant with miR-767-5p. **, *** miR-767-5p mimics vs. NC, *P* < 0.01, *P* < 0.001. **c** Transfection efficiency of MAPK4 over-expression and knocking down were determined by qRT-PCR. *** MAPK4 or sh-MAPK4 vs. control, *P* < 0.001. **d** The CCK-8 assay detects the effect of MAPK4 over-expression and knocking down on cell viability of H929 cells. ** MAPK4 or sh-MAPK4 vs. control, *P* < 0.01. **e** The CCK-8 assay detects the effect of MAPK4 over-expression and knocking down on cell viability of MM. 1S cells. *, ** MAPK4 or sh-MAPK4 vs. control, *P* < 0.05, *P* < 0.01. **f** EdU labeling assay detects the effect of MAPK4 over-expression and knocking down on cell proliferation. **, *** MAPK4 or sh-MAPK4 vs. control, *P* < 0.01, *P* < 0.001. **g** Flow cytometry detects the effect of MAPK4 over-expression and knocking down on cell cycle of H929 cells. ** MAPK4 or sh-MAPK4 vs. control, *P* < 0.01. **h** Flow cytometry detects the effect of MAPK4 over-expression and knocking down on cell cycle of MM. 1S cells. ** MAPK4 or sh-MAPK4 vs. control, *P* < 0.01. **i** IF staining detects the effect of MAPK4 over-expression and knocking down on the expression of MAPK4. **j** Western blot analysis detects the effect of MAPK4 over-expression and knocking down on the expression of MAPK4, CDK6, CDK4, Cyclin D1 and Cyclin E, p21^Cip1^

We utilized computational prediction TargetScan to identify the potential binding sites for MAPK4 mRNA on miR-767-5p. We found the 3’UTR of MAPK4 mRNA bears three potential miR-767-5p binding sites (Fig. 5a). The effect of miR-767-5p on transcription of MAPK4 was evaluated by luciferase reporter assay. Over-expression of miR-767-5p significantly decreased luciferase activity of reporter gene with wild-type MAPK4–3’UTR compared with negative control (*P* < 0.01) (Fig. 5b). However, this regulatory effect of miR-767-5p was partially suppressed when the predicted miR-767-binding site in 3’UTR of MAPK4 mRNA was mutated (Fig. 5b), suggesting other exactly binding site between miR-767-5p and MAPK4 needs further research.

In order to explore the functional role of MAPK4 on MM, plasmids highly expressed MAPK4 (MAPK4) and knockdown ofMAPK4 by shRNA (sh-MAPK4) were constructed and the efficiency of both plasmids was tested by qRT-PCR, which showed a significant increment for MAPK4 (*P* < 0.001) and reduction for sh-MAPK4 (*P* < 0.0001), respectively (Fig. 5c). The CCK8 assay demonstrated that NCI-H929 (Fig. 5d) and MM.1S cells (Fig. 5e) transfected with sh-MAPK4 significantly increased cell viability, while MAPK4 decreased cell viability (*P* < 0.01). EdU staining also showed promotion in the proliferation of MM cells by sh-MAPK4 while inhibition in the proliferation by MAPK4 (Fig. 5f). Flow cytometry of NCI-H929 (Fig. 5g) and MM.1S (Fig. 5h) cell cycle also confirmed that MAPK4 inhibited cell cycle and blocks cell cycle at G1 stage, while sh-MAPK4 promoted cell cycle progression. These data suggested that MAPK4 inhibits the cell progression of MM. IF (Fig. 5i) and Western blot (Fig. 5j) showed an increment of protein expression for MAPK4 and reduction for sh-MAPK4. Western blot also showed that MAPK4 decreased the expression of CDK4, Cyclin D1 and Cyclin E, while increased p21^Cip1^(Fig. 5j), thus blocking cell cycle at G1 stage. Sh-MAPK4 increased the expression of CDK4, Cyclin D1 and Cyclin E, while decreased p21^Cip1^(Fig. 5 j), thus promoting cell progression, confirming the inhibition ability of cell progression for MAPK4.

### MiR-767-5p promotes progression of MM by inhibiting MAPK4

**Fig. 6:**
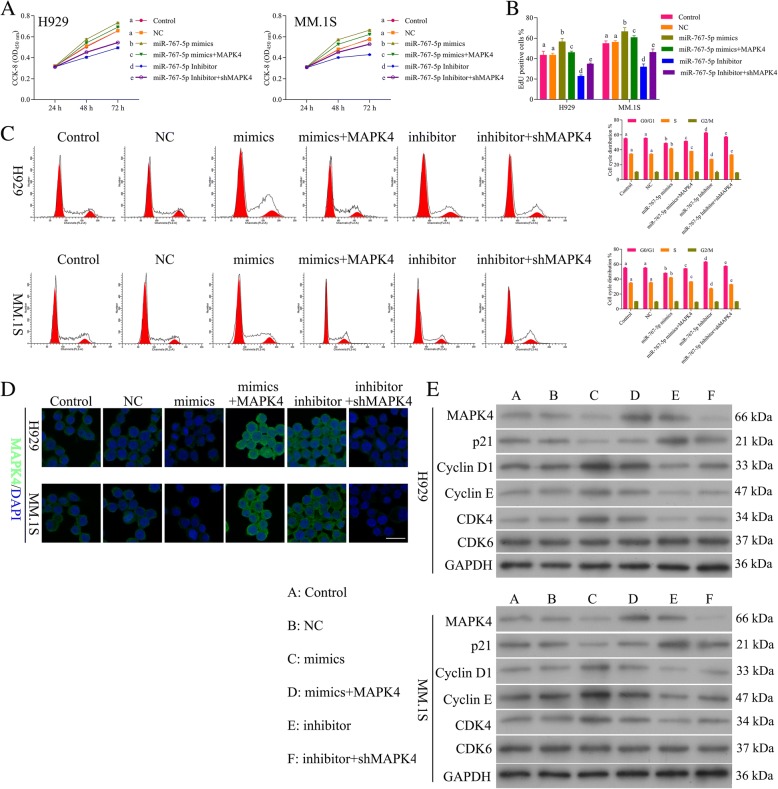
MiR-767-5p promotes progression of MM by inhibiting MAPK4. **a** The CCK8 assay detects the effect of MAPK4 and sh-MAPK4, miR-767-5p mimics and inhibitor on cell viability of H929 and MM. 1S cells. **b** EdU labeling assay detects the effect of MAPK4 and sh-MAPK4, miR-767-5p mimics and inhibitor on cell proliferation. **c** Flow cytometry detects the effect of MAPK4 and sh-MAPK4, miR-767-5p mimics and inhibitor on cell cycle. **d** IF staining detects the effect of MAPK4 and sh-MAPK4, miR-767-5p mimics and inhibitor on the expression of MAPK4. **e** Western blot analysis detects the effect of MAPK4 and sh-MAPK4, miR-767-5p mimics and inhibitor on the expression of MAPK4, CDK6, CDK4, Cyclin D1 and Cyclin E, p21^Cip1^.Different letters in the figure indicate statistically significant differences (*P* < 0.05)

In order to evaluate whether miR-767-5p promoted progression of MM by specifically targeting MAPK4, we examined four groups of MM cell models: miR-767-5p mimics, miR-767-5p mimics+MAPK4 over-expression, miR-767-5p inhibitor, miR-767-5pinhibitor + sh-MAPK4. The cell viability (Fig. 6a) and proliferation (Fig. 6b) showed increase by miR-767-5p mimics and decrease by addition of MAPK4, decrease by miR-767-5p inhibitor and increase by addition of sh-MAPK4. Flow cytometry of the cell cycle also confirmed that miR-767-5p inhibitor inhibited cell cycle and blocks cell cycle at G1 stage, while addition of sh-MAPK4 promoted cell cycle progression (Fig. 6c). Moreover, miR-767-5p mimics promoted cell cycle while inhibited by addition of MAPK4 (Fig. 6c). These data suggested that miR-767-5p promoted progression of MM by inhibiting MAPK4. IF (Fig. 6d) and Western blot (Fig. 6e) demonstrated down-regulation of MAPK4 by miR-767-5p mimics, while up-regulation by addition of MAPK4, up-regulation by inhibitor, while down-regulation by addition of sh-MAPK4. Western blot of proteins involved in cell cycle (Fig. 6e) also confirmed the promotion ability of miR-767-5p on MM through targeting MAPK4.

### Circ_0000190/miR-767-5p/MAPK4 axis on the cell progression of MM

**Fig. 7:**
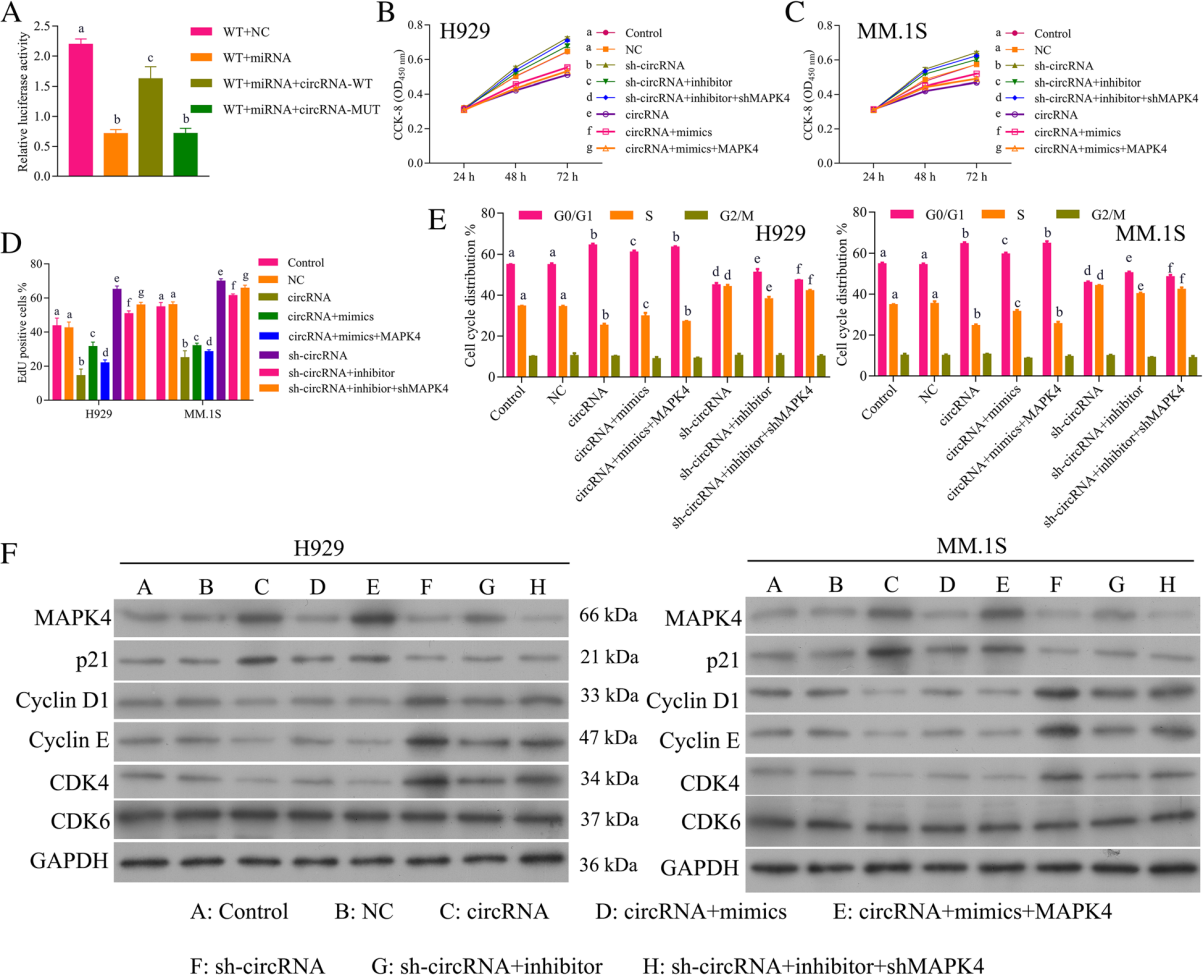
Circ_0000190/miR-767-5p/MAPK4 axis on the cell progression of MM. **a** Luciferase reporter assay of circ_0000190/miR-767-5p/MAPK4 axis. **b** The CCK8 assay detects the effect of circ_0000190 and sh- circ_0000190, miR-767-5p mimics and inhibitor, MAPK4 and shMAPK4 on cell viability of H929 cells. **c** The CCK8 assay detects the effect of circ_0000190 and sh-circ_0000190, miR-767-5p mimics and inhibitor, MAPK4 and shMAPK4 on cell viability of MM. 1S cells. **d** EdU labeling assay detects the effect of circ_0000190 and sh- circ_0000190, miR-767-5p mimics and inhibitor, MAPK4 and shMAPK4 on cell proliferation. **e** Flow cytometry detects the effect of circ_0000190 and sh-circ_0000190, miR-767-5p mimics and inhibitor, MAPK4 and shMAPK4 on cell cycle. **f** Western blot analysis detects the effect of circ_0000190 and sh-circ_0000190, miR-767-5p mimics and inhibitor, MAPK4 and shMAPK4 on the expression of MAPK4, CDK6, CDK4, Cyclin D1 and Cyclin E, p21^Cip1^. Different letters in the figure indicate statistically significant differences (*P* < 0.05)

Results above showed that circ_0000190 inhibits progression of MM by inhibiting miR-767-5p and miR-767-5p promotes progression of MM by inhibiting MAPK4. Then we decided to detect the circ_0000190/miR-767-5p/MAPK4 axis on the cell progression of MM. Dual luciferase assay showed that miR-767-5p mimics significantly decreased luciferase activity of reporter gene with wild-type MAPK4 3’UTR compared with negative control (*P* < 0.001) (Fig. 7a). However, this regulatory effect of miR-767-5p was partially suppressed by the addition of wild-type circ_0000190 3’UTR (Fig. 7a). Moreover, mutant circ_0000190 3’UTR significantly decreased luciferase activity of MAPK4, just as miR-767-5p mimics, confirming circ_0000190/miR-767-5p/MAPK4 axis.

We examined six groups of MM cell models: circ_0000190 over-expression, circ_0000190 over-expression+miR-767-5p mimics, circ_0000190 over-expression+miR-767-5p mimics+MAPK4 over-expression, sh-circ_0000190, sh-circ_0000190 + miR-767-5pinhibitor, sh-circ_0000190 + miR-767-5pinhibitor + sh-MAPK4. The cell viability of NCI-H929 (Fig. 7b) and MM.1S cells (Fig. 7c) and cell proliferation (Fig. 7d) assays showed decrease by circ_0000190 and increase by addition of miR-767-5p mimics, while further addition of MAPK4 over-expression decreased the cell viability and proliferation. Sh-circ_0000190 increased the cell viability (Fig. 7b, c) and proliferation (Fig. 7d**)** and decreased by the addition of miR-767-5p inhibitor, further addition of sh-MAPK4 could restore the decrease in cell viability and proliferation by miR-767-5p inhibitor. Flow cytometry of the cell cycle also confirmed that circ_0000190 inhibited cell cycle and blocks cell cycle at G1 stage and addition of miR-767-5p promoted cell cycle progression, while further addition of MAPK4 over-expression inhibited the cell cycle as circ_0000190 over-expression (Fig. 7e). Sh-circ_0000190 promoted the cell cycle (Fig. 7e) and inhibited by the addition of miR-767-5p inhibitor, further addition of sh-MAPK4 could restore the inhibition in cell cycle by miR-767-5p inhibitor. These data suggested that progression of MM was tightly regulated by circ_0000190/miR-767-5p/MAPK4 axis. Western blot (Fig. 7f) and IF (Fig. 7c) demonstrated up-regulation of p21^Cip1^ while down-regulation of CDK4, Cyclin D1 and Cyclin E by circ_0000190 and even more by addition of MAPK4 over-expression, while weakened by addition of miR-767-5p mimics, in contrast to sh-circ_0000190, sh-MAPK4 and miR-767-5p inhibitor.

### Circ_0000190 inhibits growth of human MM tumor in murine xenograft models

**Fig. 8:**
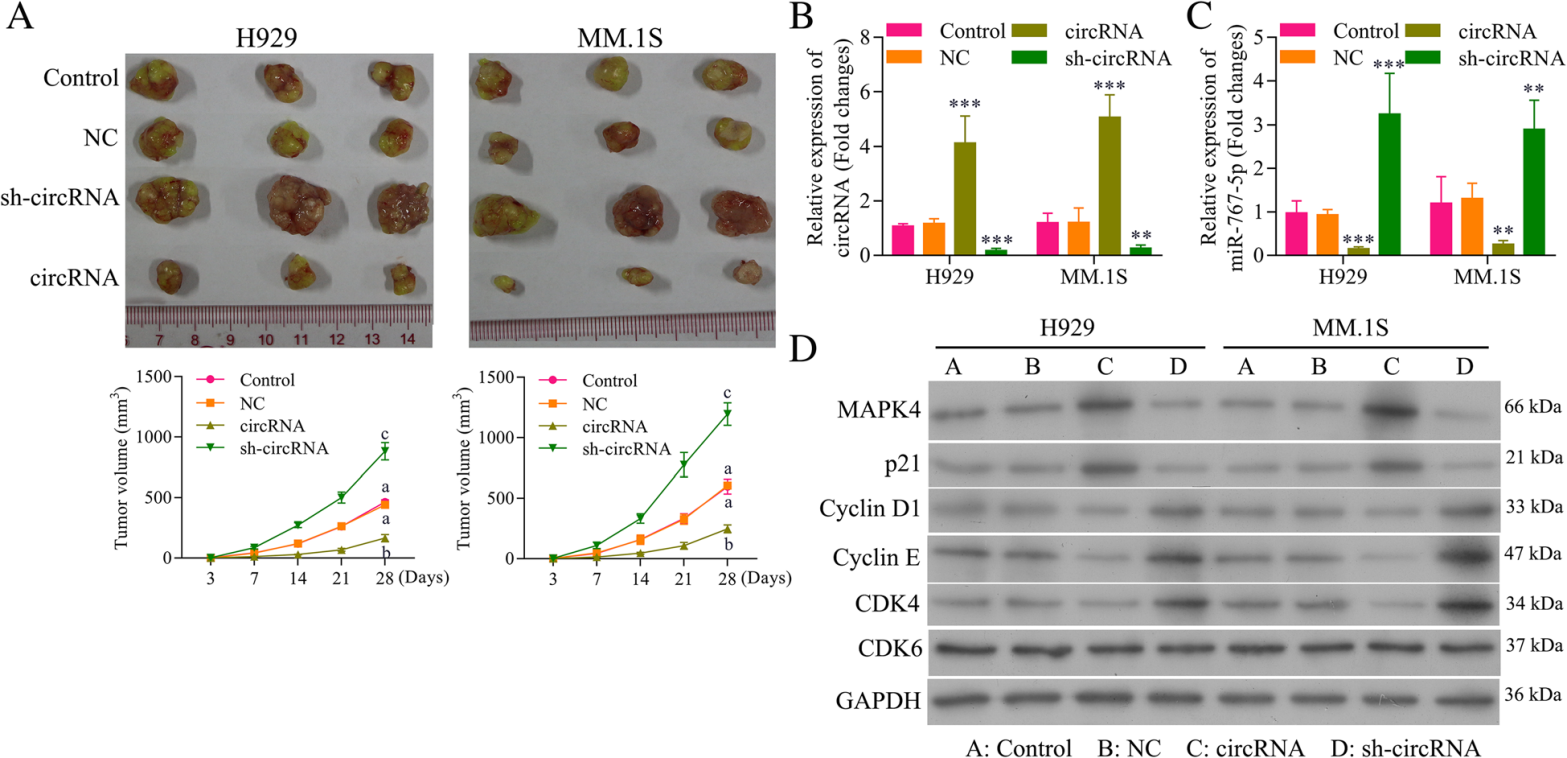
Circ_0000190 inhibits growth of human MM tumor in murine xenograft models. **a** Effect of subcutaneously injection of MM.1S and NCI-H929 cells transtected with circ_0000190 over-expression or sh-circ_0000190 on the tumor growth. **b** qRT-PCR analysis ofcirc_0000190 in MM.1S and NCI-H929 tumors models mice transfected with circ_0000190 over-expression or sh-circ_0000190. **, *** circRNA or sh-circRNA vs. control, *P* < 0.01, *P* < 0.001. **c** qRT-PCR analysis of miR-767-5p in MM.1S and NCI-H929 tumors models mice transfected with circ_0000190 over-expression or sh-circ_0000190. **, *** circRNA or sh-circRNA vs. control, *P* < 0.01, *P* < 0.001. **d** Western blot analysis of MAPK4, CDK6, CDK4, Cyclin D1 and Cyclin E, p21^Cip1^ in tumors subcutaneously injected with MM.1S and NCI-H929 cells transtected with circ_0000190 over-expression or sh-circ_0000190.Different letters in the figure indicate statistically significant differences (*P* < 0.05)

The therapeutic potential of administering circ_0000190 over-expression or sh-circ_0000190 plasmid was investigated in NOD/SCID mice-bearing both MM.1S and NCI-H929 cells subcutaneous tumors. Intratumoral injection of circ_0000190 over-expression inhibited MM tumor growth by exceed 60% compared to control animals, whereas sh-circ_0000190 promoted tumor growth more than doubled (Fig. 8a). These results confirmed the antitumoral effect of circ_0000190 in an MM tumor model. Treatment of cultured MM.1S and NCI-H929 cells with circ_0000190 over-expression resulted in an increase in expression of circ_0000190 and a reduction in expression of miR-767-5p in contrast to the effect of sh-circ_0000190 (Fig. 8b), indicating that circ_0000190 is biologically active in myeloma cells. The in vivo expression of MAPK4 protein was also detect, showing up-regulation by circ_0000190 and down-regulation by sh-circ_0000190 (Fig. 8c). Consistent with cell experiments, circ_0000190 over-expression decreased the expression of CDK4, Cyclin D1 and Cyclin E, while increased p21^Cip1^ in MM mice model, thus blocking cell cycle at G1 stage (Fig. 8d). However, sh- circ_0000190 increased the expression of CDK4, Cyclin D1 and Cyclin E, while decreased p21^Cip1^, thus promoting cell progression (Fig. 8d). Taken together, circ_0000190 demonstrated significant antitumoral activity in MM mice model.

## Discussion

**Fig. 9:**
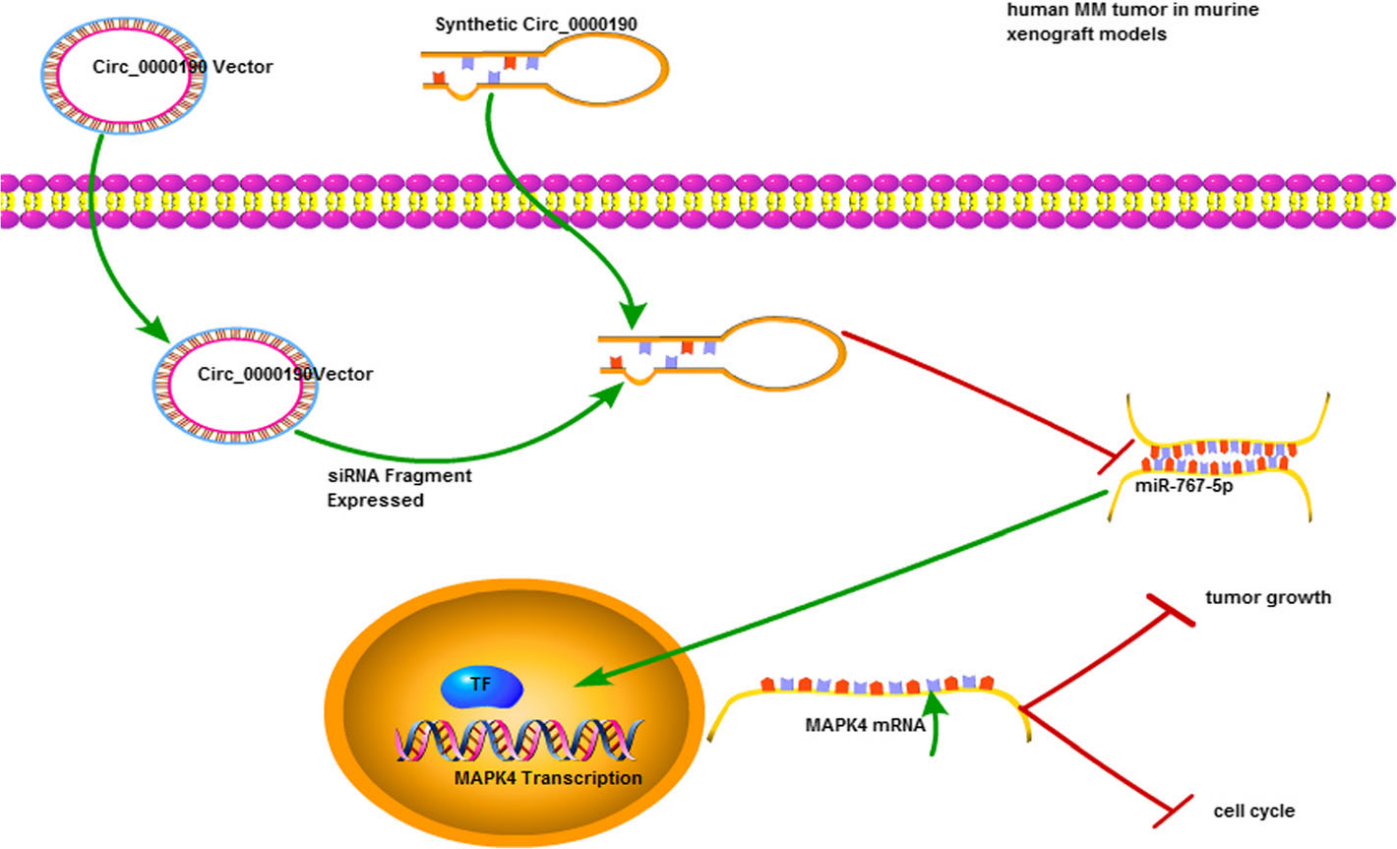
Mechanism diagram

First found in virus [36], circRNAs were then found from splicing pre-mRNA or introns [37, 38]. Extensively expressed in human cells, the unique circular structure of circRNAs protect from degradation with excellent stability [39]. Due to the aforementioned advantages, circRNAs may serve as ideal diagnostic biomarkers for cancer superior to miRNAs and lncRNAs [40, 41]. In this study, we uncovered that circRNA circ_0000190 was down- regulated in the MM tissue and peripheral blood, as well as MM cell lines for the first time. The dysregulated expression was significantly associated with prognosis survival rates of MM patients, suggesting the potential value of circ_0000190 as a biomarker or therapeutic target for MM diagnosis. To the best of our knowledge, this is the first study determining circ_0000190 inhibits tumor progression in MM via specifically targeting miR-767-5p/MAPK4 (Fig. 9). This was also confirmed in murine xenograft MM mice models. To no one’s surprise, researches have shown that cicrRNAs were found to be associated with metastasis of tumors and increased positive diagnosis rate [20, 42].

Increasing evidences have revealed relation between aberrant expression of circRNAs and tumor progression, and circRNAs functioned as an miRNA sponge. For example, circRNA_100290 was markedly up-regulated in oral squamous cell carcinoma tissue and had the binding sites for miR-29, and the knockdown of circRNA_100290 significantly inhibits the proliferative ability of cells [43]. Interference of up-regulation of circ_0000064 in lung cancer significantly blocked cell cycle progression [44]. These researches indicate that circRNAs might participate in the occurrence and development of tumors. In the present study, we found that circ_0000190 exerted antitumoral role in MM tumorigenesis in both cell model and mice model. Over-expression ofcirc_0000190 significantly decreased cell viability and proliferation, inhibited cell cycle and blocks cell cycle at G1 stage with down-regulation of CDK6, CDK4, Cyclin D1 and Cyclin E, while up-regulation p21^Cip1^.These results indicated that circ_0000190 may serve a cancer-suppressive role in MM progression.

MiR-767-5p has been predicted and validated as target of circ_0000190 using bioinformatics analysis and luciferase reporter assay. Many researched have indicated the functional role of miR-767-5p in different tumors [45–48]. MiR-767 is over-expressed in human melanoma and promoted cell proliferation as a tumor promoter gene [45]. To explore the in-depth regulatory mechanism within circ_0000190 and miR-767-5p, we performed functional experiments to test the interaction between them on progression of MM cell lines. Firstly, a negative correlation between circ_0000190 and miR-767-5p was found in both human MM tissue and plasma. Secondly, miR-767-5p mimics significantly increased cell viability and proliferation of MM cells, promoted cell cycle progression with down-regulation of CDK6, CDK4, Cyclin D1 and Cyclin E, while up-regulation of p21^Cip1^, suggesting the promotion ability of cell progression for miR-767-5p. Lastly, the decreasing cell viability and proliferation, the inhibition of cell cycle caused by circ_0000190 could be recovered by addition of miR-767-5p mimics, demonstrating that circ_0000190 exerted antitumoral role on MM cells via negatively targeting miR-767-5p. Nevertheless, due to the specific configuration and ambiguous function, the more deep-going functional mechanism of circ_0000190 involved in the MM tumorigenesis is still inconclusive and needs to be further studied.

MAPK4, members of signaling pathways involved in the development of MM, has been identified as a tumor suppressor that is down-regulated and in various adenocarcinomas or cancers, such as pancreatic adenocarcinoma [49], breast and prostate cancer [50]. Therefore, aberrant circ_0000190/miR-767-5p may be associated with the regulation of MAPK4, thus regulating cell biological functions of MM. Similarly, MAPK4 has been predicted and validated as target of miR-767-5p using bioinformatics analysis and luciferase reporter assay. It was demonstrated that the level of MAPK4 in MM cell lines was markedly increased, The expression of MAPK4 in MM.1S and NCI-H929 cells treated with circ_0000190 was dramatically decreased and the over-expression ofcirc_0000190 significantly enhanced the level of MAPK4,revealing the regulatory role of circ_0000190 on MAPK4 expression. Functional assay showed the inhibition ability of MAPK4 on cell progression of MM and miR-767-5ppromoted progression of MM through inhibiting MAPK4. In general, the present study demonstrated that circ_0000190 functioned as miR-767-5p sponges, removing the inhibitory effect of miR-767-5p on its target MAPK4, and further regulating the expression of MAPK4, which have been shown in other circRNAs/miRNAs/target axis [51, 52].

## Conclusion

In conclusion, down-regulated circ_0000190 is associated with progression in MM and over-expression of circ_0000190 significantly decreased the proliferation, viability of MM cells. Additionally, miR-767-5p/MAPK4 may serve an important role in these regulations as the downstream target. Understanding the regulatory mechanism of circ_0000190 in MM could lead to the identification of useful clinical biomarker or indicator. Overall, circ_0000190 may serve as a prospective biomarker and a promising target for MM.

## Additional files

Additional file 1: **Figure S1.** The intra cellular localization of circular RNA by the means of single-molecule RNA fluorescence in situ hybridization (smFISH). circ_0000190 was mainly located in the cytoplasm of MM cells. (TIF 11720 kb)

Additional file 2: **Figure S2.** The expression of circ_0000190 in different multiple myeloma cell lines (TIF 153 kb)

Additional file 3: **Figure S3.** Tunel stain was performed to detect the effect of circ_0000190 on MM cell apoptosis. (TIF 25031 kb)

## Abbreviations

ANOVA: One-way analysis of variance
ceRNAs: Competitive endogenous RNAs
circRNAs: Circular RNAs
DS: Durie-salmon
EdU: 5-Ethynyl-2′-Deoxyuridine
FITC: Fluorescein isothiocyanate
gDNA: Genome DNA
ISS: International staging system
lncRNAs: Long non-coding RNAs
MAP K4: Mitogen-activated protein kinase 4
miRNAs: MicroRNAs
MM: Multiple myeloma
OS: Overall survival
PFS: Progression-free
PI: Propidium iodide
qRT-PCR: Quantitative Real Time Polymerase Chain Reaction
