# Supplementary figures and images for: CircRNA circ_0000190 inhibits the progression of multiple myeloma through modulating miR-767-5p/MAPK4 pathway
Source: J Exp Clin Cancer Res. 2019 Feb 6;38:54. doi: 10.1186/s13046-019-1071-9 (PMC6364482; doi:10.1186/s13046-019-1071-9)

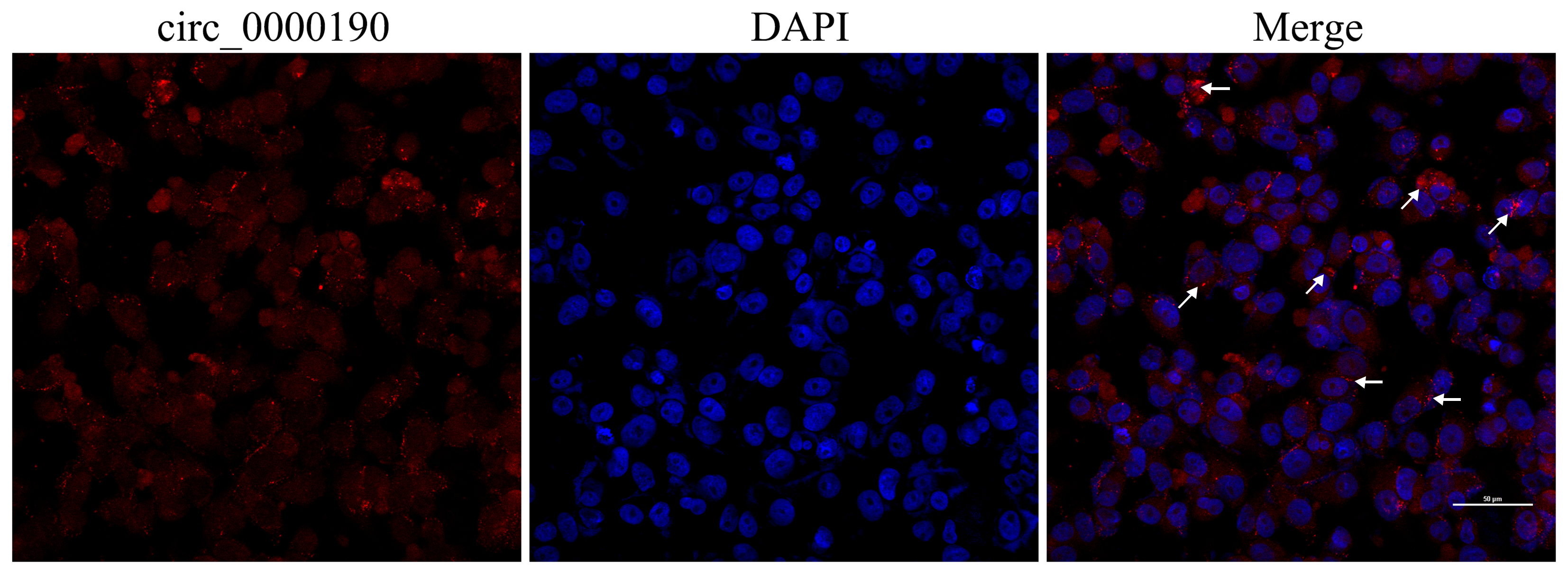

Supplement: Supplementary file 1 — Figure S1. The intra cellular localization of circular RNA by the means of single-molecule RNA fluorescence in situ hybridization (smFISH). circ_0000190 was mainly located in the cytoplasm of MM cells. (TIF 11720 kb) [file 13046_2019_1071_MOESM1_ESM.tif]

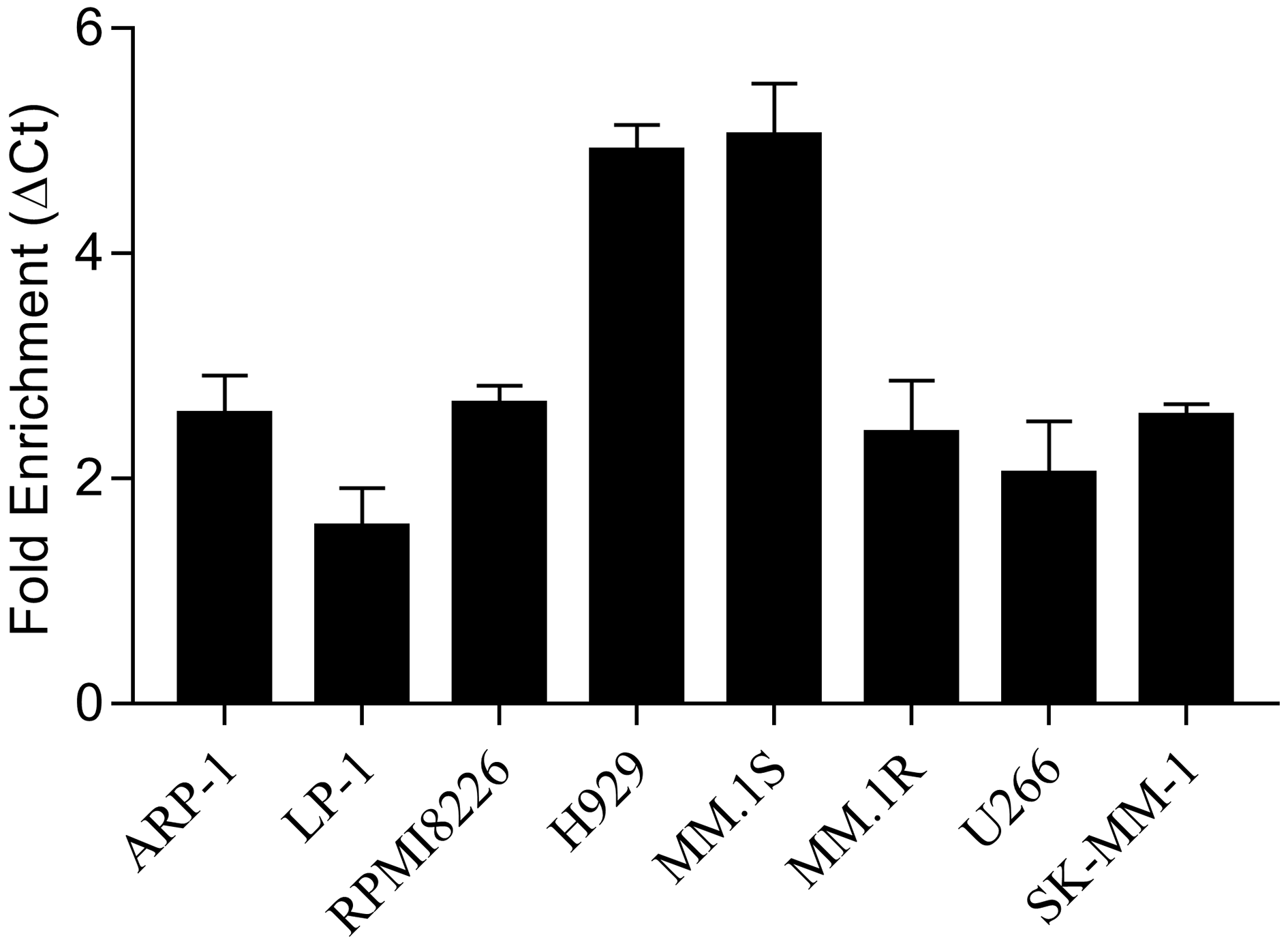

Supplement: Supplementary file 2 — Figure S2. The expression of circ_0000190 in different multiple myeloma cell lines (TIF 153 kb) [file 13046_2019_1071_MOESM2_ESM.tif]

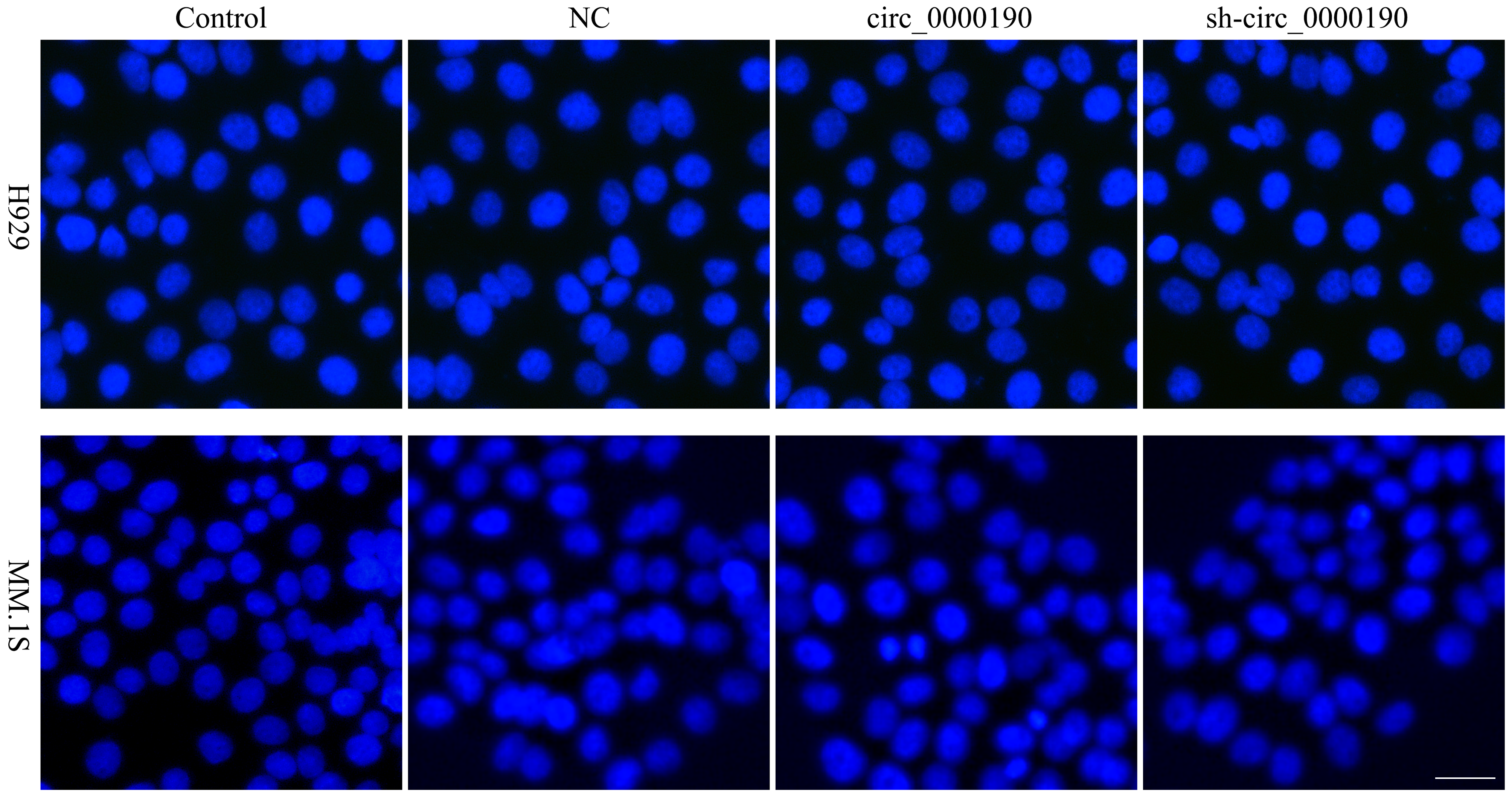

Supplement: Supplementary file 3 — Figure S3. Tunel stain was performed to detect the effect of circ_0000190 on MM cell apoptosis. (TIF 25031 kb) [file 13046_2019_1071_MOESM3_ESM.tif]
